# Correspondence on “Robotic radical hysterectomy for cervical cancer: current trends and controversies”

**DOI:** 10.7150/jca.114989

**Published:** 2025-08-22

**Authors:** Agnieszka Rychlik, Maria Bedyńska, Denis Querleu

**Affiliations:** 1 Department of Gynecologic Oncology, National Research Institute of Oncology, Warsaw, Poland.; 2 UOC Ginecologia Oncologica, Dipartimento di Scienze della Salute della Donna, del Bambino e di Sanità Pubblica, Fondazione Policlinico Universitario Agostino Gemelli IRCCS, Rome, Italy.

Dr Kim and colleagues must be commended for addressing the issue of outcomes of robotic assistance in the minimal invasive management of cervical cancer.

The background of this letter is to respectfully challenge their conclusion that robotic radical hysterectomy (RRH) which should rather be named robotic-assisted laparoscopic radical hysterectomy “*offers significant perioperative benefits, including reduced blood loss, shorter hospital stays, and fewer complications*”. This indeed holds if the term of comparison is laparotomy, but not if the term of comparison is laparoscopy. Indeed, the only available randomized study addressing this question is negative 2. ROBOGYN-1004 (ClinicalTrials.gov, NCT01247779) was a multicenter, phase III, superiority randomized trial that compared robotic-assisted laparoscopy and conventional laparoscopy in patients with gynecologic cancer. The primary endpoint was incidence of severe complications. Robotic assisted surgery was not found superior to laparoscopic radical hysterectomy (LRH) in this regard. Robotic assisted laparoscopy was not better in terms of any of the secondary endpoints: oncological outcomes and other perioperative results (conversion rate, and blood loss).

The choice to exclusively focus on robotic approach leads Dr Kim *et al.* to complete their conclusive statement by “*without compromising oncologic outcomes such as overall survival and progression-free survival*” while not mentioning laparoscopic surgery as an option. Finally, they conclude that “*based on this systematic review, RRH is a safe and effective alternative to abdominal approach for early-stage cervical cancer*” ignoring the absence of evidence of superiority of robotic assistance in oncologic outcomes compared with traditional laparoscopy.

This choice is not supported by any evidence. Indeed, in the minimal invasive surgery group of the LACC trial, the only available randomized controlled trial in this field 3 4.6 years survival rates were similar in the robotic and laparoscopic groups (87.2 versus 87.0, respectively). In addition, in four well-conducted meta-analyses, no evidence of superiority of robotic assistance was found 4-6. In the metanalysis by Nitecki *et al.*, including authors of the LACC trial, this point is addressed in Figures 1 and 2, which did not show any impact on survival of the proportion of robotic assistance in the included trials 4. The metanalysis by Hwang included 3121 patients from 20 studies 5. Although most of the included studies were retrospective and nonrandomized, oncological efficacy was comparable between RRH and LRH. In a 2023 metanalysis excluding robotic cases, the detrimental effect of minimal invasive surgery on survival after radical hysterectomy disappears 6. In addition, the RECOURSE study did not find any difference in recurrence free or overall survival outcomes between laparoscopy and RRH in endometrial cancer 7.

As a result of the marketing efforts of the industry, one may observe the increasing use of an expensive tool with no demonstrated benefit and at the same time a regrettable loss of laparoscopic skills and training in academic institutions. The statement that “*robotic surgery has advantages for complex surgical procedures in the deep and narrow pelvic cavity* “is not supported by any high-level patient-oriented outcome data. However, this is likely be true in the setting of obese patients, yet, like any definitive statement in medicine, this must be supported by a randomized study. This question is currently addressed in a randomized controlled study in the setting of endometrial cancer 8.
